# Supplementary material for: Effects of Waterlogging at Flowering Stage on the Grain Yield and Starch Quality of Waxy Maize
Source: Plants (Basel). 2023 Dec 29;13(1):108. doi: 10.3390/plants13010108 (PMC10780669; doi:10.3390/plants13010108)
Supplement: Supplementary file 1 [file plants-13-00108-s001.zip › plants-2677236-supplementary-main (1).pdf]

plants MDPI

Table S1. Meteorological conditions during plant growth in 2014 and 2015.

Parameter	Year	March			April			May			June			July		
		F	M	L	F	M	L	F	M	L	F	M	L	F	M	L
Temperature	2014	7	12	15	16	15	18	18	21	26	24	24	25	24	28	29
(°C)	2015	6	11	14	11	15	20	19	22	22	24	25	23	24	26	29
Rainfall	2014	28	48	17	4	102	22	1	60	4	22	18	35	100	58	90
(mm)	2015	10	90	0	40	25	20	20	45	35	100	100	250	20	75	20
Sunlight	2014	48	70	54	94	24	56	83	65	90	54	42	32	38	21	58
(h)	2015	40	30	90	35	60	90	55	65	50	50	55	15	45	30	75

Note: F, M, L are the first, middle, and last ten-day of a month.

Table S2. Effects of waterlogging at the flowering stage on the flour pasting property of waxy maize.

Year	Hybrid	Water	Granule Size (μm)	Peak1/Peak2	Relative Crystallinity (%)
2014	SYN5	CK	13.54 d	3.38 d	19.65 d
		WS	14.25 bc	5.24 a	18.37 e
	YN7	CK	14.49 b	4.86 b	15.64 f
		WS	14.01 c	3.79 c	19.97 d
2015	SYN5	CK	14.05 c	3.55 d	37.45 a
		WS	15.59 a	3.81 c	29.77 b
	YN7	CK	15.31 a	2.81 e	27.78 c
		WS	12.57 e	2.75 e	29.85 b

Mean value in the same column followed by different letters is significantly different ($p < 0.05$). SYN5, Suyunuo5; YN7, Yunuo7; CK, control; WS, waterlogging.
